# Supplementary material for: Tension can directly suppress Aurora B kinase-triggered release of kinetochore-microtubule attachments
Source: Nat Commun. 2022 Apr 20;13:2152. doi: 10.1038/s41467-022-29542-8 (PMC9021268; doi:10.1038/s41467-022-29542-8)
Supplement: Supplementary file 1 — Supplementary Information [file 41467_2022_29542_MOESM1_ESM.pdf]

Supplementary Information for de Regt et al:

Supplementary Figures 1-6

Supplementary References

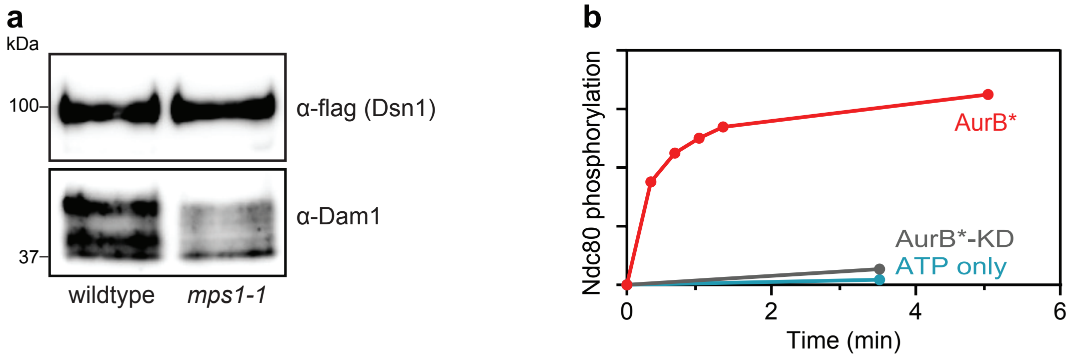

Supplementary Figure 1. Control data related to Figure 1. (**a**) Immunoblot of purified kinetochores from either wild-type (SBY8253) or *mps1-1* cells (SBY8726) showing that Mps1-1 kinetochores lack Dam1c. (**b**) Quantification of 32P incorporation into Ndc80, based on the autoradiogram shown in Figure 1b. The red points (AurB*) are from the time course, which is shown in lanes three through seven of the gel in Figure 1b. The blue point (ATP only) is from a 3.5-min mock treatment, shown in the second to last lane of the gel in Figure 1b. The grey point (AurB*-KD) is from a 3.5-min treatment with kinase-dead mutant, shown in the last lane of the gel in Figure 1b. Units on the vertical axis are arbitrary. Source data are provided as a Source data file.

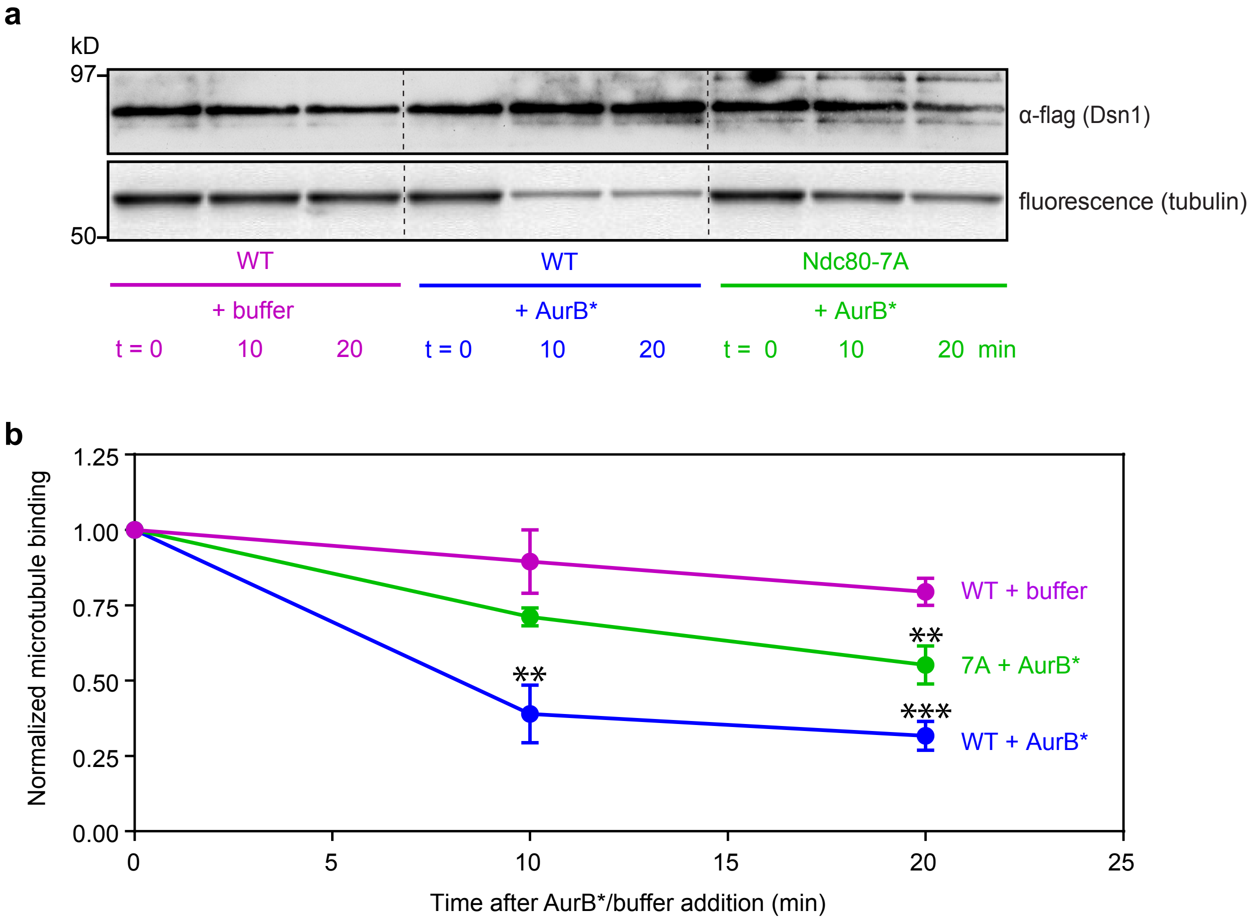

Supplementary Figure 2. Phosphorylation of physiologically relevant sites accelerates release of microtubules from kinetochores. (**a**) Wild-type (WT) kinetochores (from SBY8253) or phospho-deficient mutant Ndc80-7A kinetochores (from SBY8522) were immunoprecipitated onto anti-flag dynabeads, washed, and then incubated for five minutes with taxol-stabilized fluorescently-labeled microtubules in the presence of ATP. Buffer or 1 μM AurB* was then added, at t = 0 min, and dynabead subsamples were removed, washed and quenched at the indicated times. Components retained in each subsample were separated via SDS-PAGE and visualized by immunoblot (against Dsn1-flag) and fluorescence scan (to detect bound tubulin). Source data are provided as a Source Data file. (**b**) Tubulin fluorescence in each subsample plotted as a function of elapsed time since the addition of AurB* or buffer alone. Points represent the average band intensities from three independent experiments like that shown in (a), normalized to the intensities at t = 0. Error bars represent standard deviation. The p-values between the t = 20 min timepoints were calculated by comparing means with a two-tailed unpaired t-test with Welch's correction and are as follows: 0.0002 (WT+buffer versus WT+AurB*), 0.0077 (WT+buffer versus 7A+AurB*), 0.0084 (WT+AurB* versus 7A+AurB*).

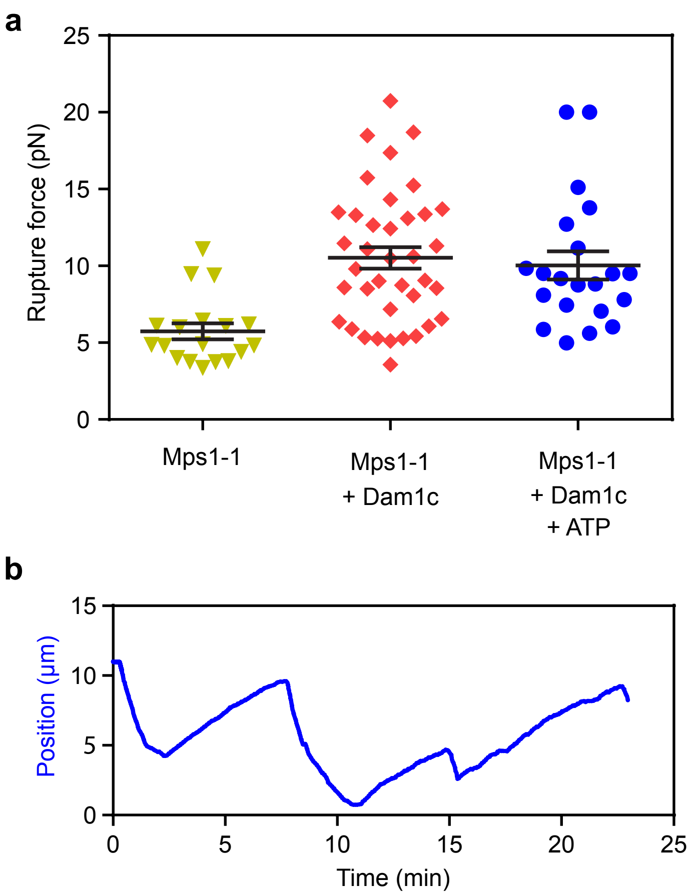

Supplementary Figure 3. Adding Dam1c strengthens Mps1-1 kinetochores. (**a**) Rupture strengths measured for Mps1-1 kinetochores using an optical force-ramp. Where indicated, exogenously purified Dam1 complex (Dam1c) and/or ATP were included in the trapping buffer. The central line represents mean and the error bars represent SEM from n = 18 individual rupture force measurements for Mps1-1, n = 38 for Mps1-1 + Dam1, and n = 21 for Mps1-1 + Dam1 + ATP. (**b**) Trace of optical trap data for an individual force-clamp event, recorded using a bead decorated with Mps1-1 kinetochores in the presence of free purified Dam1c. The relative position versus time is plotted for a single kinetochore-decorated bead as it continuously supports 5 pN of tension while tracking with the dynamic tip of an individual microtubule during tip growth and shortening. These data (a, b) were collected using a high density of kinetochores on the trapping beads (Dsn1:bead ratio, 3,300), using one biochemical preparation of kinetochores and one preparation of Dam1c, to eliminate any possible confounding effects due to prep-to-prep variability. Source data are provided as a Source Data file.

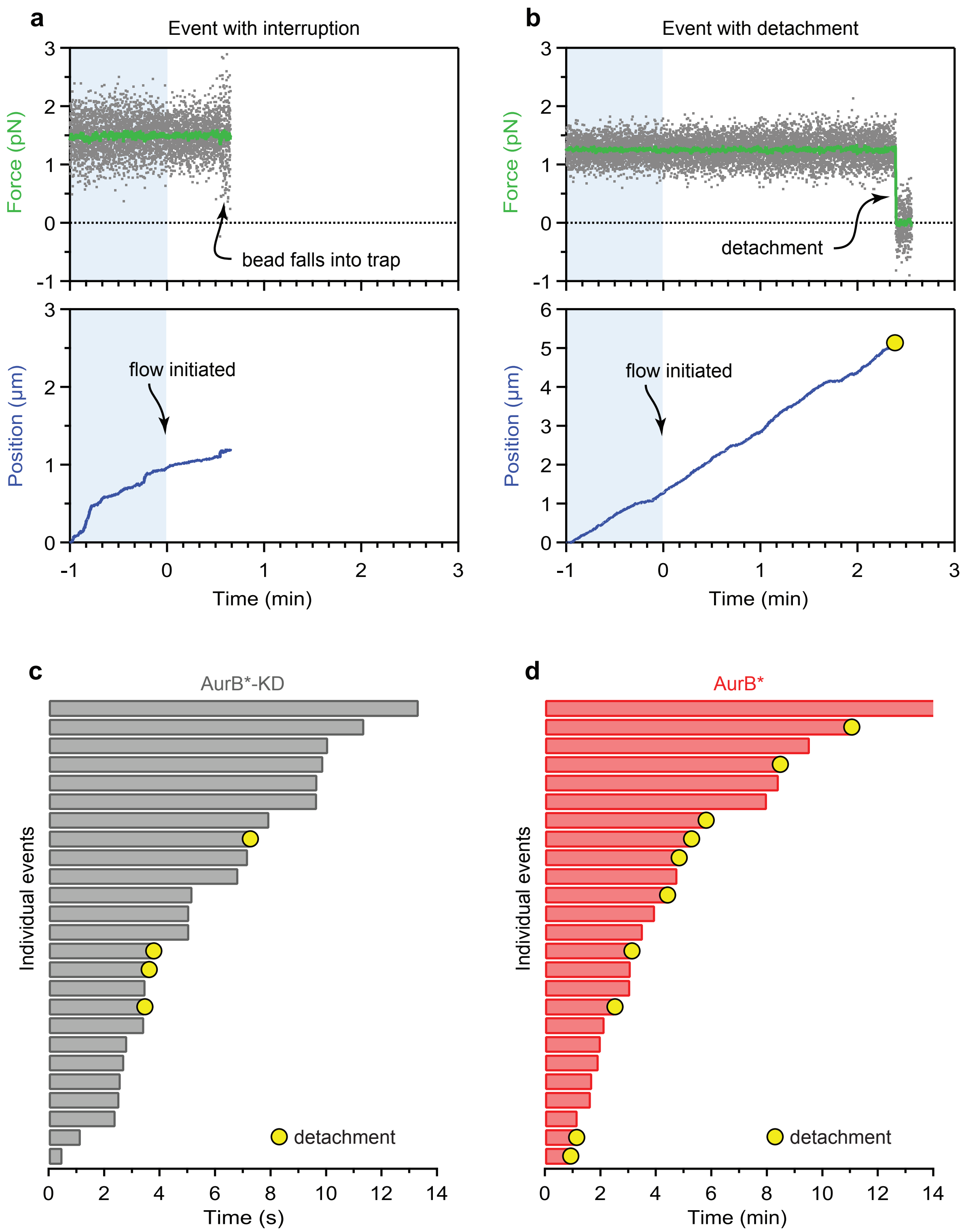

Supplementary Figure 4. Analysis of individual events recorded using the optical trap flow assay. (**a**, **b**) Example records of optical trap data for two individual events. Gray points show the force applied and green traces show the same data after smoothing. Blue traces show relative positions of the kinetochore-decorated beads over time. Kinase introduction occurred at time 0; blue shading indicates data recorded before kinase introduction. (a) shows an event that ended when a second bead became caught in the laser trap. (b) shows an event that ended in a detachment. The yellow circle indicates when a detachment occurred. (**c**, **d**) Durations of twenty-five randomly selected kinetochore-microtubule tip attachments supporting ~1 pN of tension in the presence of either 0.5 µM kinase-dead mutant AurB*-KD (c) or 0.5 µM active AurB* (d). Events that ended in detachment are marked with yellow circles. These data (a - d) were collected using a high density of kinetochores on the trapping beads (Dsn1:bead ratio, 3,300). Source data are provided as a Source Data file.

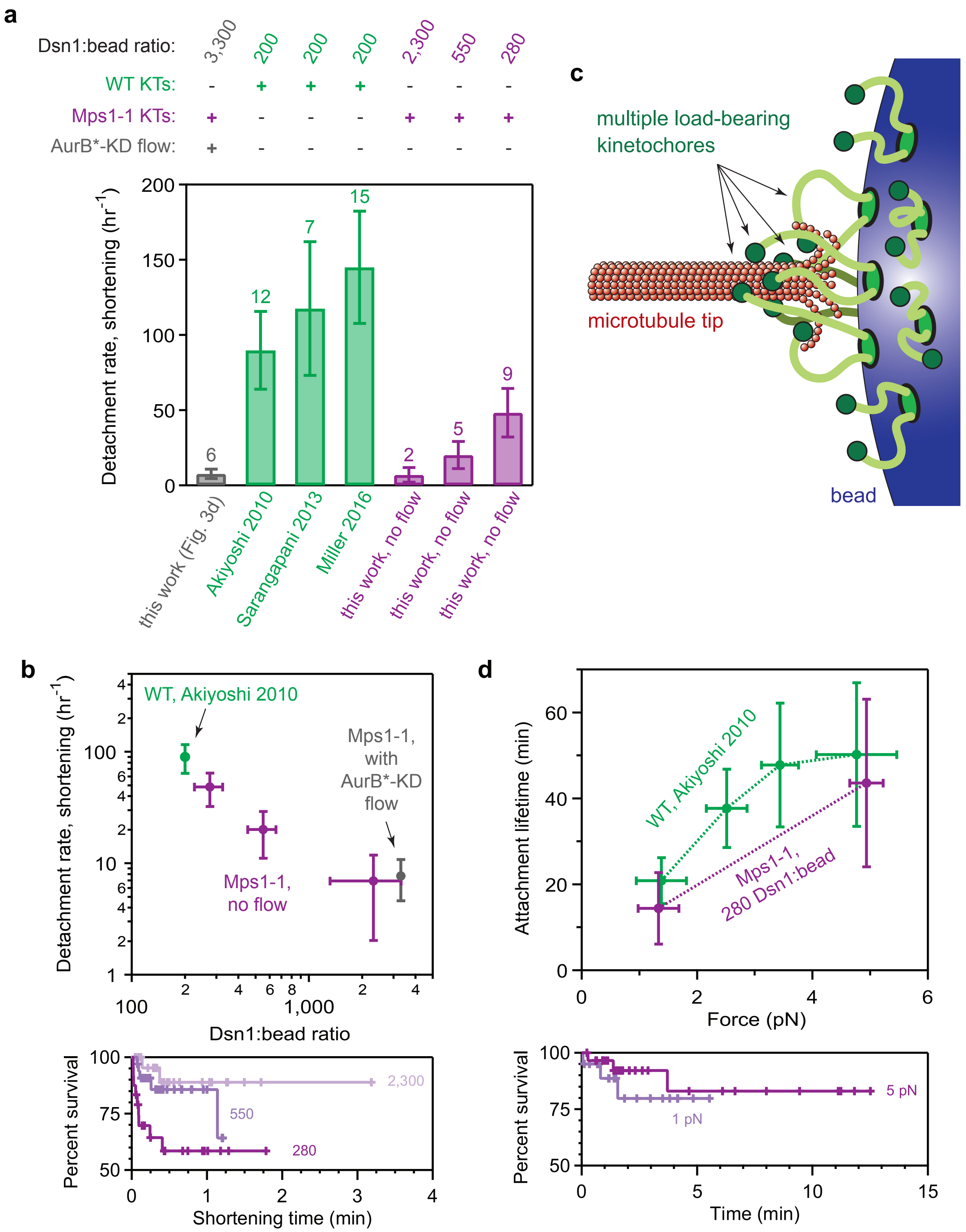

Supplementary Figure 5. A high density of kinetochores on beads suppresses detachment, suggesting applied loads are shared by multiple kinetochores. (**a**, **b**) Detachments from shortening microtubule tips were unusually rare during initial flow assay experiments, which used a high density of Mps1-1 kinetochores on the trapping beads (AurB*-KD, grey bar; Dsn1:bead ratio, 3,300) relative to previous measurements that used beads decorated much more sparsely with wild-type kinetochores (green bars; Dsn1:bead ratio, 200; from [Akiyoshi 2010; Sarangapani 2013; Miller 2016]). Reducing the density of Mps1-1 kinetochores on the beads restored the higher detachment rates (purple bars; Dsn:bead ratios indicated). Values above bars in (a) indicate numbers of detachment events for each condition. Error bars represent uncertainty due to Poisson statistics. (**c**) If multiple kinetochores share the applied load, then the load per kinetochore can be low even when the total load is high. (**d**) With sparser decoration on the trapping beads (Dsn1:bead ratio, 280), the Mps1-1 kinetochores exhibit a catch bond-like increase in attachment stability with force, consistent with previous studies using wild-type kinetochores (Dsn1:bead ratio, 200). Some load-sharing might still occur even at the sparsest decoration densities, since detachment rates during shortening remain sensitive to the Dsn1:bead ratio (as shown in b). The data plotted in purple in (a, b) and (d) were collected using one biochemical preparation of kinetochores and one preparation of Dam1c, to eliminate any possible confounding effects due to prep-to-prep variability. Source data, including numbers of detachments, observation times, rate estimates, and statistical comparisons are provided as a Source Data file.

a

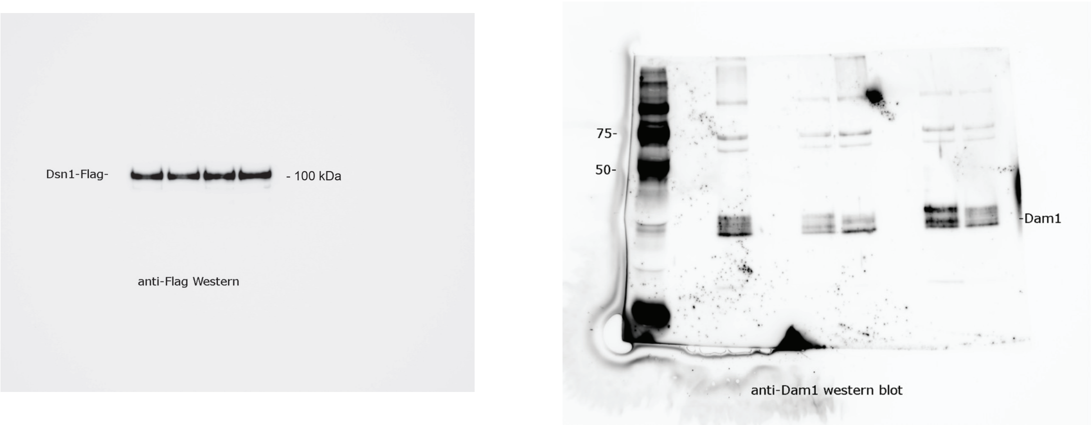

b

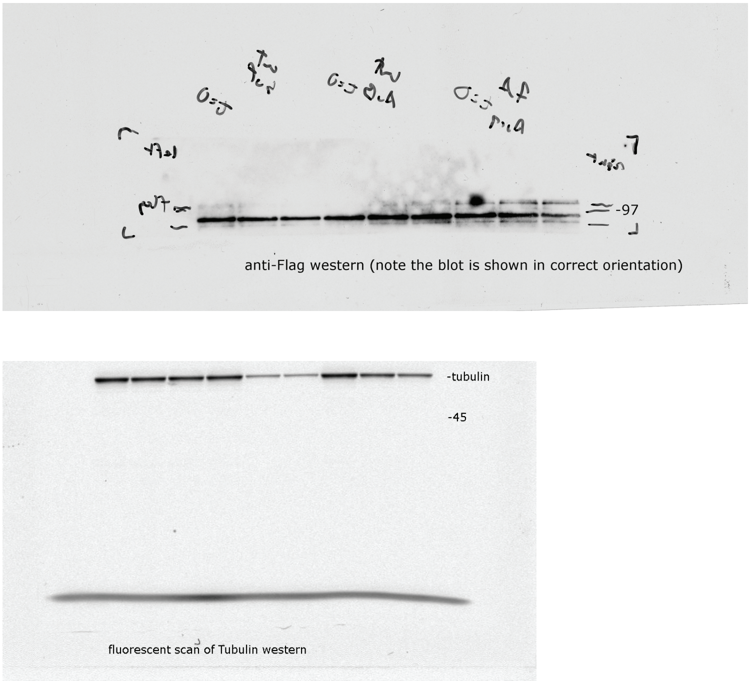

Supplementary Figure 6. Raw data related to Supplementary Figures 1 and 2a. (**a**) The entire immunoblots for the data in Supplementary Figure 1 are shown. (**b**) The raw immunoblots for the data in Supplementary Figure S2 are shown.

Supplementary References

1. Akiyoshi B, Sarangapani KK, Powers AF, Nelson CR, Reichow SL, Arellano-Santoyo H, Gonen T, Ranish JA, Asbury CL, Biggins S. Tension directly stabilizes reconstituted kinetochore-microtubule attachments. *Nature* **468**, 576-579 (2010).

2. Sarangapani KK, Akiyoshi B, Duggan NM, Biggins S, Asbury CL. Phosphoregulation promotes release of kinetochores from dynamic microtubules via multiple mechanisms. *Proc Natl Acad Sci U S A* **110**, 7282-7287 (2013).

3. Miller MP, Asbury CL, Biggins S. A TOG Protein Confers Tension Sensitivity to Kinetochore-Microtubule Attachments. *Cell* **165**, 1428-1439 (2016).
