# Supplementary material for: Tension can directly suppress Aurora B kinase-triggered release of kinetochore-microtubule attachments
Source: Nat Commun. 2022 Apr 20;13:2152. doi: 10.1038/s41467-022-29542-8 (PMC9021268; doi:10.1038/s41467-022-29542-8)
Supplement: Supplementary file 3 — Reporting Summary [file 41467_2022_29542_MOESM3_ESM.pdf]

nature portfolio

Corresponding author(s): Sue Biggins, Charles L. Asbury

Last updated by author(s): January 12, 2022

Reporting Summary

Nature Portfolio wishes to improve the reproducibility of the work that we publish. This form provides structure for consistency and transparency in reporting. For further information on Nature Portfolio policies, see our Editorial Policies and the Editorial Policy Checklist.

Statistics

For all statistical analyses, confirm that the following items are present in the figure legend, table legend, main text, or Methods section.

n/a	Confirmed	
☐	☒	The exact sample size (*n*) for each experimental group/condition, given as a discrete number and unit of measurement
☒	☐	A statement on whether measurements were taken from distinct samples or whether the same sample was measured repeatedly
☐	☒	The statistical test(s) used AND whether they are one- or two-sided<br>*Only common tests should be described solely by name; describe more complex techniques in the Methods section.*
☒	☐	A description of all covariates tested
☒	☐	A description of any assumptions or corrections, such as tests of normality and adjustment for multiple comparisons
☐	☒	A full description of the statistical parameters including central tendency (e.g. means) or other basic estimates (e.g. regression coefficient) AND variation (e.g. standard deviation) or associated estimates of uncertainty (e.g. confidence intervals)
☐	☒	For null hypothesis testing, the test statistic (e.g. *F*, *t*, *r*) with confidence intervals, effect sizes, degrees of freedom and *P* value noted<br>*Give P values as exact values whenever suitable.*
☒	☐	For Bayesian analysis, information on the choice of priors and Markov chain Monte Carlo settings
☒	☐	For hierarchical and complex designs, identification of the appropriate level for tests and full reporting of outcomes
☒	☐	Estimates of effect sizes (e.g. Cohen's *d*, Pearson's *r*), indicating how they were calculated

Our web collection on statistics for biologists contains articles on many of the points above.

Software and code

Policy information about availability of computer code

Data collection	Custom software written in Labview (National Instruments) was used for laser trap instrument control and data collection. We currently run it in Labview 2018 and the source code is publicly available at https://github.com/casbury69/laser-trap-control-and-data-acquisition
Data analysis	Custom software written in Igor Pro (Wavemetrics) was used for laser trap data analysis . We currently run it in Igor Pro 8 and the source code is publicly available at https://github.com/casbury69/laser-trap-data-analysis.

For manuscripts utilizing custom algorithms or software that are central to the research but not yet described in published literature, software must be made available to editors and reviewers. We strongly encourage code deposition in a community repository (e.g. GitHub). See the Nature Portfolio guidelines for submitting code & software for further information.

Data

Policy information about availability of data

All manuscripts must include a data availability statement. This statement should provide the following information, where applicable:

- Accession codes, unique identifiers, or web links for publicly available datasets
- A description of any restrictions on data availability
- For clinical datasets or third party data, please ensure that the statement adheres to our policy

All source data are provided as an Excel document entitled Source Data, with sheets corresponding to each relevant figure. This file includes full scans of all the gels and blots, from Figures 1b, 2a, 2c, S1a, and S2a, and all the raw source data used to generate the rate estimates of Figures 3b, 3c, 3d, 4, and S5.

March 2021

Field-specific reporting

Please select the one below that is the best fit for your research. If you are not sure, read the appropriate sections before making your selection.

☒ Life sciences ☐ Behavioural & social sciences ☐ Ecological, evolutionary & environmental sciences

For a reference copy of the document with all sections, see nature.com/documents/nr-reporting-summary-flat.pdf

Life sciences study design

All studies must disclose on these points even when the disclosure is negative.

Sample size	No statistical methods were used to predetermine sample size. We based the sample size on prior experiments in which measurements were repeated until at least ten detachments were observed or until the protein preparations were exhausted. The trap data were recorded using one set of biochemical preparations (of kinetochores, Dam1c, and AurB*), to eliminate any possible confounding effects due to prep-to-prep variability
Data exclusions	No data were excluded from analysis.
Replication	The majority of experiments were successfully replicated. There was one condition where we potentially had load sharing of kinetochores on beads that was not replicated when we diluted the number of kinetochores and that is explained in the manuscript.
Randomization	The experiments were not randomized because there was only one treatment group of kinetochores used. These were randomly treated with or without ATP to perform optical trapping.
Blinding	The investigators were not blinded to allocation during experiments and outcome assessment. Many of the optical trapping measurements were performed during the pandemic when only one person was allowed to enter the laboratory. These restrictions made it impractical to try to keep the samples blinded.

Reporting for specific materials, systems and methods

We require information from authors about some types of materials, experimental systems and methods used in many studies. Here, indicate whether each material, system or method listed is relevant to your study. If you are not sure if a list item applies to your research, read the appropriate section before selecting a response.

Materials & experimental systems

n/a	Involved in the study	
☐	☒	Antibodies
☒	☐	Eukaryotic cell lines
☒	☐	Palaeontology and archaeology
☒	☐	Animals and other organisms
☒	☐	Human research participants
☒	☐	Clinical data
☒	☐	Dual use research of concern

Methods

n/a	Involved in the study	
☒	☐	ChIP-seq
☒	☐	Flow cytometry
☒	☐	MRI-based neuroimaging

Antibodies

Antibodies used	1. Anti-Ndc80 polyclonal N-terminus antibody, generated in Arshad Desai's lab (UCSD); used at 1:10,000<br>2. Anti-Flag M2 antibody, Sigma M2 F1804; used at 1:3,000<br>3. Anti-Tubulin antibodies, EMD Millipore, clone YL1/2; MAB1864; used at 1:1,000<br>4. Anti-Dam1 polyclonal antibodies, from anti-Dam1 serum that was custom generated by Pacific Immunology (Ramona CA); used at 1:5,000<br>5. All secondary antibodies used in this study were purchased from GE Healthcare and the catalog numbers are: Anti-mouse (NA931), anti-rabbit (NA934) and anti-rat (NA935). All were used at 1:1,000.

Validation

1. Anti-Ndc80 N-terminus antibody was generated in Arshad Desai's lab and has been validated using yeast strains where the endogenous Ndc80 protein was epitope tagged with another epitope to confirm that the protein shifted and that the bands matched
2. Anti-Flag M2 antibody has been used extensively in our laboratory and validated in many publications by comparing yeast strain lysates where proteins are Flag tagged or untagged on westerns
3. Anti-Tubulin antibodies have been verified by millipore as described on website (https://www.emdmillipore.com/US/en/product/Anti-alpha-Tubulin-Antibody-clone-YOL1-34,MM_NF-CBL270-I?ReferrerURL=https%3A%2F%2Fwww.google.com%2F#).
4. Anti-Dam1 antibodies were from anti-Dam1 serum that was custom generated by Pacific Immunology and previously verified in our laboratory as described in Gutierrez et al., Current Biology 30, p.4491-4499, 202.
5. Secondary antibodies have been used extensively in our laboratory and validated by the same methods as the primary antibodies as well as with negative controls lacking primary antibodies to confirm specificity.
